# Laparoscopy-assisted endoscopic ultrasound-guided gastroenterostomy for the treatment of gastric outlet obstruction

**DOI:** 10.1055/a-2761-0359

**Published:** 2026-02-17

**Authors:** Kangli Guo, Yongkang Zhang, Yijun Zhan, Nannan Wu, Yundong You, Xiaoying Han

**Affiliations:** 1 74731 Gastrointestinal Disease Diagnosis and Treatment Center, Xiangyang Central Hospital, Affiliated Hospital of Hubei University of Arts and Science, Xiangyang, China; 2 74731 Department of Medical Administration, Xiangyang Central Hospital, Affiliated Hospital of Hubei University of Arts and Science, Xiangyang, China

Gastric outlet obstruction (GOO) prevents patients from eating normally and impairs their quality of life. The main goal of treating GOO is to relieve the obstruction, allowing patients to resume normal oral intake. At present, there are various treatment options for GOO, such as surgical gastroenterostomy and endoscopic duodenal stenting. Endoscopic ultrasound-guided gastroenterostomy (EUS-GE) is a new option for relieving GOO. It has advantages such as minimal trauma and high success rates 1 . This article shares the treatment process of a patient who still had GOO after undergoing surgical gastroenterostomy, providing a reference for the treatment of patients with GOO complicated by anatomical structure changes.

**Fig. 1:**
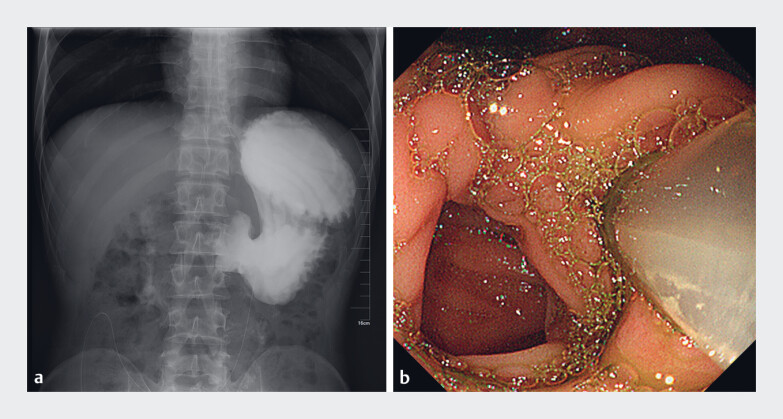
Preoperative iodine-based contrast radiography and gastroscopy. **a** Retention of iodine contrast medium in the stomach due to stenosis at the gastroenterostomy anastomosis. **b** Stenosis of the efferent loop inlet with an indwelling nasojejunal feeding tube.

**Fig. 2:**
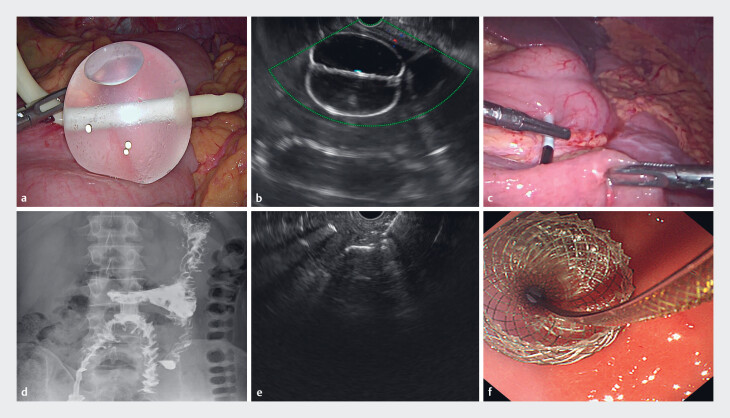
Laparoscopic and endoscopic procedures. **a** Under laparoscopy, a water-inflated balloon was placed on the greater curvature of the gastric body to locate the puncture site. **b** Localization of the water-inflated balloon under endoscopic ultrasound (EUS). **c** Laparoscopically assisted puncture of the target intestinal segment. **d** Release of the distal flange of the stent under EUS guidance. **e** Release of the proximal flange in the stomach. **f** Three months postoperatively, iodine-based contrast radiography showed that the iodine contrast medium passed through the gastroenterostomy stent smoothly.

A 35-year-old man with GOO caused by recurrent duodenal ulcer underwent surgical gastroenterostomy over 3 months ago. However, the patient still could not take food after the operation. Upper gastrointestinal iodine-based contrast radiography indicated stenosis of the gastroenterostomy anastomotic stoma, so a nasojejunal feeding tube was placed for enteral nutrition. The patient was re-admitted to the hospital 1 month later. Upper gastrointestinal iodine-based contrast radiography and ultra-fine gastroscopy revealed the complete obstruction of the efferent loop ( Fig. 1 **a, b** ). After a multidisciplinary discussion, it was considered that the patient had an anatomical variation, making the EUS-GE inapplicable. Additionally, the patient was in poor general condition and faced the risk of anastomotic fistula following surgery. Finally, it was decided to perform laparoscopy-assisted EUS-GE ( Video 1 ). The procedure was as follows ( Fig. 2 **a–e** ): Laparoscopic adhesiolysis was performed using an ultrasonic scalpel. Under the dual guidance of EUS and laparoscopy, the gastric puncture site was localized. Guided by EUS, the Axios stent (15 × 10 mm Hot AXIOS; Boston Scientific; Marlborough, Massachusetts, USA) was advanced through the gastric wall into the abdominal cavity. Laparoscopically, the small intestinal segment of the efferent loop was lifted to the anterior wall of the lower gastric body, and the stent was guided into the small intestinal lumen using an atraumatic forceps. The stent was deployed and its deployment was checked under the monitoring of EUS and laparoscopy. The operation duration is 15 minutes. During over 3 months of follow-up, the stent functioned well ( Fig. 2 **f** ) and the patient could resume a regular diet, with a weight gain of 3 kg.

Endoscopic ultrasound-guided gastroenterostomy assisted by laparoscopy for gastric outlet obstruction.Video 1

In this case, the patient had previously undergone gastroenterostomy, resulting in altered anatomical structures. Laparoscopic adhesiolysis and assistance in precisely locating the target intestinal segment for puncture ensured the successful implementation of EUS-GE, preventing incorrect stent placement. This constitutes an effective and safe therapeutic measure.

Endoscopy_UCTN_Code_TTT_1AS_2AK
